# Recurrent knee arthritis diagnosed as juvenile idiopathic arthritis with a 10-year asymptomatic period after arthroscopic synovectomy: a case report

**DOI:** 10.1186/1752-1947-7-166

**Published:** 2013-06-27

**Authors:** Atsushi Teramoto, Kota Watanabe, Yuichiro Kii, Miki Kudo, Hidenori Otsubo, Takuro Wada, Toshihiko Yamashita

**Affiliations:** 1 Department of Orthopedic Surgery, Sapporo Medical University School of Medicine, South1 West16, Chuo-ku, Sapporo 060-8543, Japan

## Abstract

**Introduction:**

Juvenile idiopathic arthritis is a chronic inflammatory disease associated with arthritis of unknown etiology that begins before the age of 16 and persists for longer than 6 weeks. The frequency of recurrence after arthroscopic synovectomy in patients with oligoarthritis juvenile idiopathic arthritis was reported to be lower than that in patients with polyarthritis. However, recurrence in cases of oligoarthritis after arthroscopic knee synovectomy was shown to be 67% in one recent study and, furthermore, a shorter period free from recurrence was also reported after synovectomy. Here we report a child who suffered recurrent knee arthritis with a 10-year asymptomatic period after arthroscopic synovectomy.

**Case presentation:**

A 12-year-old Japanese girl presented with normal birth and developmental history. At the age of 2 years she experienced joint swelling in both knees. Her symptoms continued and arthroscopic synovectomy was eventually performed. During the operation, rice bodies and thickening of the synovial membrane were observed; however, no definitive diagnosis was made. After a 10-year asymptomatic period, knee joint swelling recurred on one side without any cause. Arthroscopic synovectomy was beneficial in reducing the symptoms and in diagnosis.

**Conclusions:**

Children who suffer prolonged joint swelling have a risk of juvenile idiopathic arthritis. Even if the symptoms heal and no definite diagnosis is made at the first treatment, informed consent is needed to make the patients understand the future risk of recurrent arthritis after even lengthy asymptomatic periods.

## Introduction

Juvenile idiopathic arthritis (JIA) is a chronic inflammatory disease associated with arthritis of unknown etiology that begins before the age of 16 and persists for longer than 6 weeks [1]. JIA has several disease categories and pathologies. These categories include systemic, polyarthritis, and oligoarthritis [2]. The frequency of recurrence after arthroscopic synovectomy in patients with oligoarthritis was reported to be lower than that in patients with polyarthritis [3]. However, recurrence in cases of oligoarthritis after arthroscopic knee synovectomy was shown to be 67% in one recent study [4]. Furthermore, a shorter period free from recurrence was also reported after synovectomy [4].

The diagnosis of JIA is essentially a clinical one. Without specific clinical and laboratory findings, the differential diagnosis of JIA is extensive and diagnosis can be difficult.

Here we report the case of a child who suffered recurrent knee arthritis with a 10-year asymptomatic period after arthroscopic synovectomy.

## Case presentation

**Figure 1:**
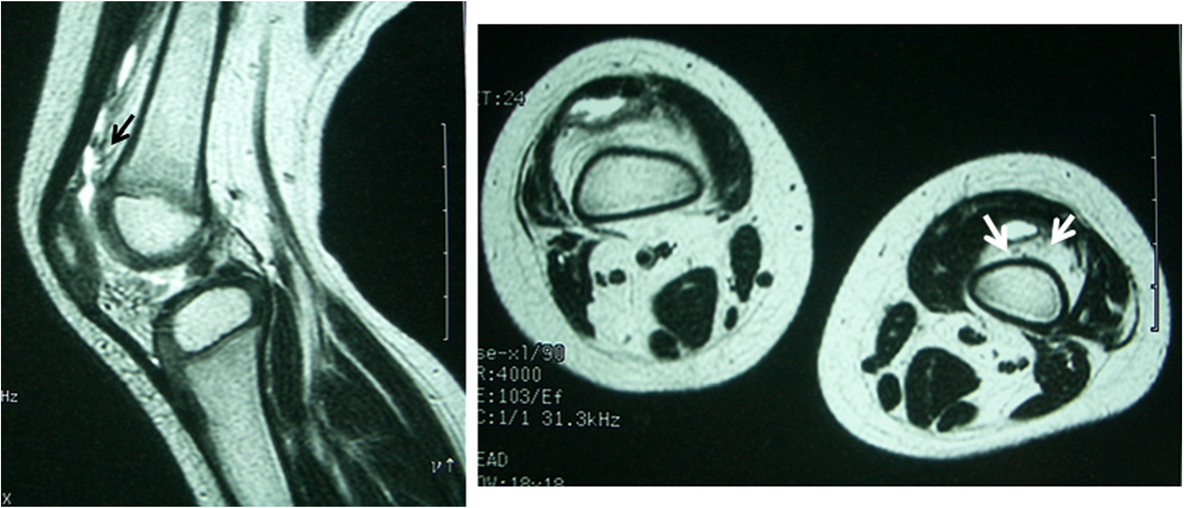
Magnetic resonance imaging (T2-weighted) at 2 years showed a small mass in her knee joint (arrows).

**Figure 2:**
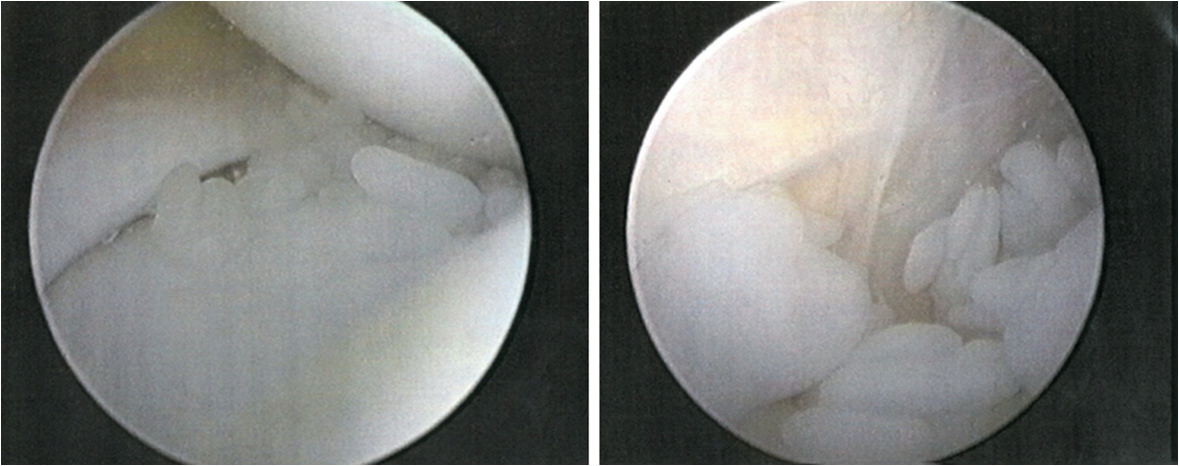
**Arthroscopic findings at 2 years showed rice bodies and thickening of the synovial membrane.** Removal of the rice bodies and synovectomy was performed for both knees.

**Figure 3:**
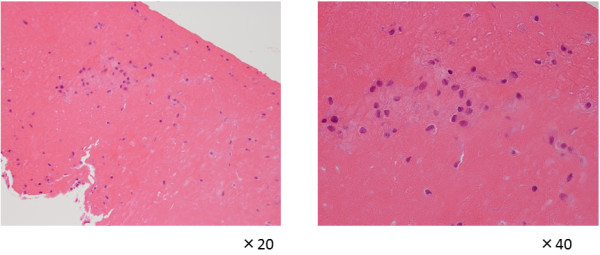
Pathological findings of the rice bodies revealed acidophilic tissues with lymphoid infiltration.

A 12-year-old Japanese girl presented with normal birth and developmental history. There was no notable family or medical past history. At 2 years old, she experienced swelling of both knee joints without any cause. There was no rest pain in either knee; however, pain on motion in the right knee and joint effusion in both knees were noted at the first examination. Full extension was observed for the right knee but flexion was limited to 130°. The left knee had a normal range of movement. Aspiration of clear yellow joint fluid showed a negative culture result. Laboratory findings showed a white blood cell (WBC) count of 10,700/μL, C-reactive protein (CRP) of 0.45mg/dL, and erythrocyte sedimentation reaction (ESR) of 21mm/hour. Magnetic resonance imaging (MRI) revealed small masses in her knee joints (Figure 1). Her symptoms were not improved after several joint aspirations. Arthroscopic synovectomy was eventually performed for both knees. During the operation, rice bodies and thickening of the synovial membrane were observed (Figure 2). The pathological findings for the rice bodies showed acidophilic tissues with lymphoid infiltration (Figure 3); however, no definite diagnosis was made. After the operation, the pain and swelling in the knees were improved. A 10-year asymptomatic period after arthroscopic synovectomy precluded the need for annual medical examinations.

**Figure 4:**
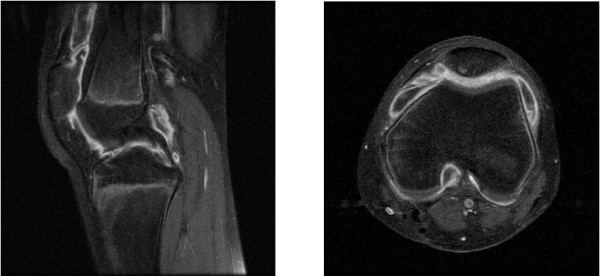
Contrast-enhanced magnetic resonance imaging (fat suppression T1-weighted) at 12 years showed joint effusion and thickening of the synovial membrane.

**Figure 5:**
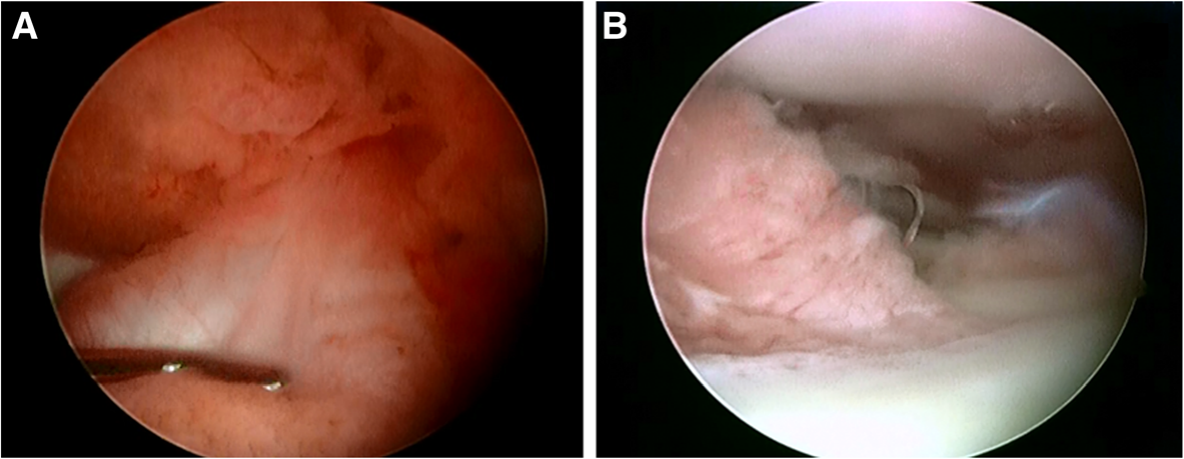
**Arthroscopic findings during synovectomy at 12 years. A**: Anterior cruciate ligament and the engorged synovial membranes. **B**: The synovial membranes in the suprapatellar pouch.

**Figure 6:**
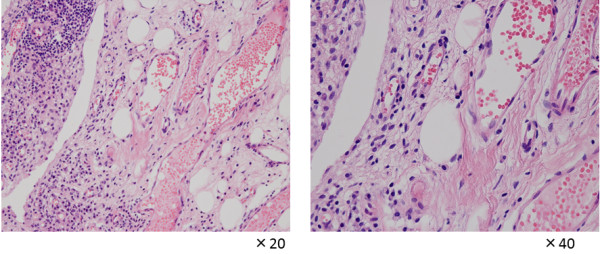
Pathological findings at 12 years revealed the presence of a villiform structure, increased blood vessels, chronic inflammatory cells, and lymphocyte infiltration.

When she was 12 years old, her left knee again showed swelling without any cause. Several joint aspirations and intra-articular injection of steroids failed to improve her symptoms. She did not experience any pain in her left knee. Full extension was observed for her knee but flexion was limited to 130°. Laboratory findings revealed a WBC count of 4,300/μL, CRP of 0.10mg/dL, ESR of 12mm/hour, rheumatoid factor (RF) of < 5IU/mL, matrix metalloproteinase-3 of 95.1ng/mL, and an antinuclear antibody (ANA) test was 1:80 positive. Contrast-enhanced MRI showed joint effusion and thickening of the synovial membrane (Figure 4). An arthroscopic synovectomy was performed for her left knee (Figure 5), and pathological findings revealed the presence of a villiform structure, increased blood vessels, chronic inflammatory cells, and lymphocyte infiltration (Figure 6). She had no uveitis, but was diagnosed with JIA and received methotrexate (MTX) medication. Since that time there has been no recurrent knee arthritis.

## Discussion

JIA is classified into three broad categories: systemic, polyarthritis, and oligoarthritis [2]. Oligoarthritis is prevalent in females, with negative RF and positive ANA results being pathognomonic [5]. Oligoarthritis has a relatively good prognosis compared with the other categories. Our patient was diagnosed with oligoarticular JIA when she was 12 years old; however, no definitive diagnosis was made when her symptoms first appeared at 2 years old. The diagnosis of JIA is made based on the child’s symptoms and the results of physical and blood examinations. It is recognized that a definite diagnosis is difficult as there is no single, definitive laboratory test for JIA. Joint effusion and numerous rice bodies were observed in the knees of our patient during her first operation. Rice body formation is not a frequently observed pathology and diseases accompanied by rice body formation in the affected joints include soft tissue tumors, such as synovial chondromatosis, or chronic arthritis (septic, tuberculous, or systemic). The existence of rice bodies is not considered to be typical of JIA. However, we think from our experience that JIA should be taken into consideration during differential diagnosis when rice bodies are identified [6,7]. We performed arthroscopic synovectomy for diagnosis at 2 years of age. Since the pathological findings for the rice bodies showed acidophilic tissues and no definite diagnosis was made, biopsy at 2 years of age might not have been helpful for the diagnosis of JIA. However, soft tissue tumors and tuberculous arthritis could be ruled out.

The general approach to treating JIA includes nonsteroidal anti-inflammatory drugs and multiple intra-articular injection of steroids as first-line therapy, and the second option is MTX or a biological product. If these are not sufficient, synovectomy might be an effective treatment option for mono-articular JIA. The surgical treatment for JIA remains controversial. Adamec *et al.*[8] reported that surgical treatment should be evaluated in cooperation with a rheumatologist after administration of an appropriate conservative therapy for at least 6 months. Dell'Era *et al.*[4] reported the outcomes of 31 knee synovectomies (six cases of oligoarthritis, 20 of polyarthritis, and five of psoriatic arthritis) in 19 children with JIA. The best results were observed in the group with oligoarthritis. They concluded that the aim of arthroscopic synovectomy was to enhance nonsurgical therapy. In our patient, no definite diagnosis was made before the second operation, although knee swelling was prolonged and repeated knee aspiration was required. Arthroscopic knee synovectomy was helpful in reducing her symptoms and in making a diagnosis of atypical recurrent JIA, which leaded to treatment with the appropriate medication. Typically, surgical intervention is not recommended until erosions are radiologically manifested. However, we think that arthroscopic synovectomy is one of the options of treatment and prevention of bone erosions for JIA. Toledo *et al.*[9] described the following: that arthroscopy made it possible to remove synovial tissue mechanically during the subacute phase of mono-articular JIA, might prevent pannus formation, and may lead to complete remission of arthritis. Medication for JIA also remains controversial. She received MTX medication due to recurrence of knee arthritis. There has been no recurrent knee arthritis, and no side effects.

The recurrence of symptoms, even in oligoarthritis, is not uncommon, with the relapse rate after synovectomy reported to be 36 to 67% [4,9]. In addition, the mean period free from recurrence was only 1.69 years [4]. Thus, surgical treatment alone may not be sufficient for the treatment of JIA.

This patient was diagnosed as oligoarticular JIA and was treated with arthroscopic synovectomy of both knees at the age of 2 years. After a 10-year asymptomatic period, arthritis recurred in one knee without any cause. Children who suffer prolonged joint swelling are at risk of JIA. Even if the symptoms heal and no definite diagnosis is made at the first treatment, informed consent is needed to make the patients understand the future risk of recurrent arthritis after even lengthy asymptomatic periods.

## Conclusions

We reported a child who suffered recurrent knee arthritis with a 10-year asymptomatic period after arthroscopic synovectomy. Informed consent is needed to make the patients understand the future risk of recurrent arthritis of JIA after even lengthy asymptomatic periods.

## Consent

Written informed consent was obtained from the patient's parent for publication of this manuscript and accompanying images. A copy of the written consent is available for review by the Editor-in-Chief of this journal.

## Competing interests

The authors declare that they have no competing interests.

## Authors’ contributions

AT was a major contributor in writing the manuscript. AT, KW, YK, MK, and HO performed the 2^nd^ surgery and several examinations of the patient and carried out the follow-up of the patient. TW performed the 1^st^ surgery and carried out the follow-up of the patient for 10 years. TY conceived of the study, participated in its design and coordination, and helped to draft the manuscript. All authors read and approved the final manuscript.

## References

[B1] Brewer EJ Jr Bass J Baum J Cassidy JT Fink C Jacobs J Hanson V Levinson JE Schaller J Stillman JS Current proposed revision of JRA criteria Arthritis Rheum 1977 20 Suppl 2 195 199 318120

[B2] Petty RE Southwood TR Baum J Bhettay E Glass DN Manners P Maldonado-Cocco J Suarez-Almazor M Orozco-Alcala J Prieur AM Revision of the proposed classification criteria for juvenile idiopathic arthritis: Durban, 1997 J Rheumatol 1998 25 1991 1994 9779856

[B3] Butbul YA Tyrrell PN Schneider R Dhillon S Feldman BM Laxer RM Saurenmann RK Spiegel L Cameron B Tse SM Silverman ED Comparison of patients with juvenile psoriatic arthritis and nonpsoriatic juvenile idiopathic arthritis: how different are they? J Rheumatol 2009 36 2033 2041 10.3899/jrheum.080674 19648305

[B4] Dell'Era L Facchini R Corona F Knee synovectomy in children with juvenile idiopathic arthritis J Pediatr Orthop B 2008 17 128 130 10.1097/BPB.0b013e32809256f2 18391810

[B5] Ravelli A Felici E Magni-Manzoni S Pistorio A Novarini C Bozzola E Viola S Martini A Patients with antinuclear antibody-positive juvenile idiopathic arthritis constitute a homogeneous subgroup irrespective of the course of joint disease Arthritis Rheum 2005 52 826 832 10.1002/art.20945 15751057

[B6] Wynne-Roberts CR Cassidy JT Juvenile rheumatoid arthritis with rice bodies: light and electron microscopic studies Ann Rheum Dis 1979 38 8 13 434952 10.1136/ard.38.1.8 PMC1000309

[B7] Chung C Coley BD Martin LC Rice bodies in juvenile rheumatoid arthritis AJR Am J Roentgenol 1998 170 698 700 10.2214/ajr.170.3.9490956 9490956

[B8] Adamec O Dungl P Kasal T Chomiak J Knee joint synovectomy in treatment of juvenile idiopathic arthritis Acta Chir Orthop Traumatol Cech 2002 69 350 356 12587496

[B9] Toledo MM Martini G Gigante C Da Dalt L Tregnaghi A Zulian F Is there a role for arthroscopic synovectomy in oligoarticular juvenile idiopathic arthritis? J Rheumatol 2006 33 1868 1872 16881093

